# Percutaneous endoscopic lumbar discectomy in lumbar disc herniation with posterior ring apophysis fracture: A case report in a 15-year-old child

**DOI:** 10.1097/MD.0000000000036213

**Published:** 2023-12-29

**Authors:** Baode Zhang, Peikai Chen, Jiaquan Zhong, Michael Kai-Tsun To, Kenneth Man-Chee Cheung, Jianbin Wu

**Affiliations:** a Department of Orthopedics and Traumatology, The University of Hong Kong – Shenzhen Hospital (HKU-SZH), Shenzhen, Guangdong, China; b School of Biomedical Sciences, The University of Hong Kong, Pok Fu Lam, Hong Kong; c Department of Orthopedics and Traumatology, The University of Hong Kong, Pok Fu Lam, Hong Kong.

**Keywords:** case report, lumbar disc herniation, pediatric, percutaneous endoscopic lumbar discectomy, posterior ring apophysis fracture

## Abstract

**Rationale:**

Lumbar disc herniation (LDH) with posterior ring apophysis fracture (PRAF) is rather rare in children, and in all age-stratified LDH patients, the incidence of RAF was 5.3% to 7.5%. Interestingly, the incidence of LDH with RAF in children (15%–32%) is several times higher than in adults, the mis-diagnosis of which may lead to delayed treatment.

**Patient concerns:**

Here, we report a 15-year-old schoolboy who suffered from sudden low back pain and radiating pain in both lower limbs after sport activities. Symptoms persisted after 3 months of conservative treatment. Computer radiography and magnetic resonance imaging indicated central disc herniation with PRAF at L4-5.

**Diagnosis:**

LDH with PRAF.

**Interventions:**

The herniated disc and epiphyseal fragments were successfully excised by the percutaneous endoscopic lumbar discectomy minimal-invasive technique.

**Outcomes:**

Surgery was successful. Symptoms were immediately relieved postoperatively with a wound of only about 7.0 mm. Discharged on the next day. No perioperative complications occurred. Moreover, the imaging and clinical outcomes were also more satisfactory during the post-operative 15 months outpatient follow-up.

**Lessons:**

Pediatric LDH with PRAF is extremely uncommon, and there is a lack of training among physicians for such cases, which may lead to delayed diagnosis and treatment. Once a diagnosis for LDH with PRAF is established, percutaneous endoscopic lumbar discectomy is a safe and effective minimally invasive treatment to be considered, and we hope that this technique can provide more assistance in the future.

## 1. Introduction

Lumbar disc herniation (LDH) is a spinal orthopedic condition commonly associated with the aging population. It is rarely reported in adolescents and children, where they generally account for 0.4% to 15.4% all herniation cases.^[1,2]^ Pediatric LDH with fracture or separation of ring apophysis is even rarer. In all age-stratified LDH patients, the incidence of RAF was 5.3% to 7.5%.^[3–5]^ Interestingly, the incidence of LDH with RAF in children (15%–32%) is several times higher than in adults.^[6–8]^

Intervertebral disc degeneration and/or spinal mechanical overload are the 2 major factors in adult LDH.^[2]^ Conversely, sports injury or genetic factors, rather than chronic degeneration, were thought to be more relevant in pediatric LDH.^[2,9–16]^ It has been reported that 30% to 60% of symptomatic LDH in children and adolescents have a history of trauma prior to the onset of pain.^[17]^ The youngest child case reported was a 13-month-old male infant whose condition may have been caused by a fall in a standing position.^[18]^ The first documented lumbar discectomy in children can be traced back to a 12-year-old female gymnast reported by Wahren in 1945.^[19]^ Here, we report a case of a rare pediatric LDH with posterior ring apophysis fracture (PRAF), successfully treated by percutaneous endoscopic lumbar discectomy (PELD).

## 2. Case report

**Figure 1.**
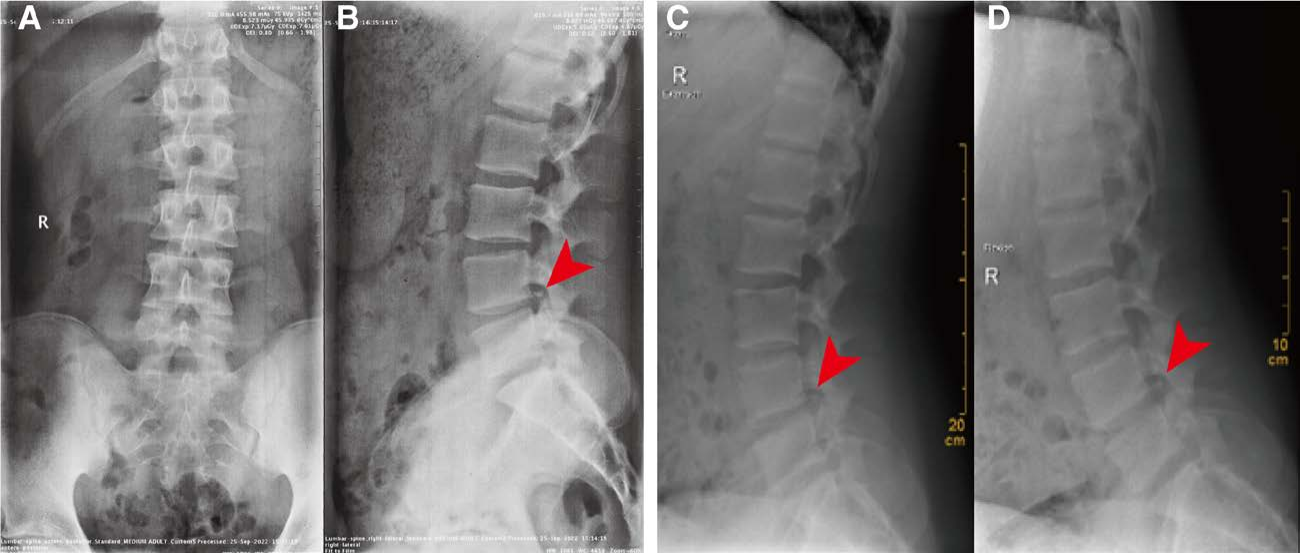
Pre-operative lumbar spine anteroposterior, lateral, dynamic position views by X-ray. (A and B) Lumbar spine is anteroposterior and lateral (L-spine AP &LAT). (C and D) Lumbar spine dynamic position. The L4-5 level vertebral space was narrowed, fragments of PRAF at the lower L4 vertebral body were clearly visible.

**Figure 2.**
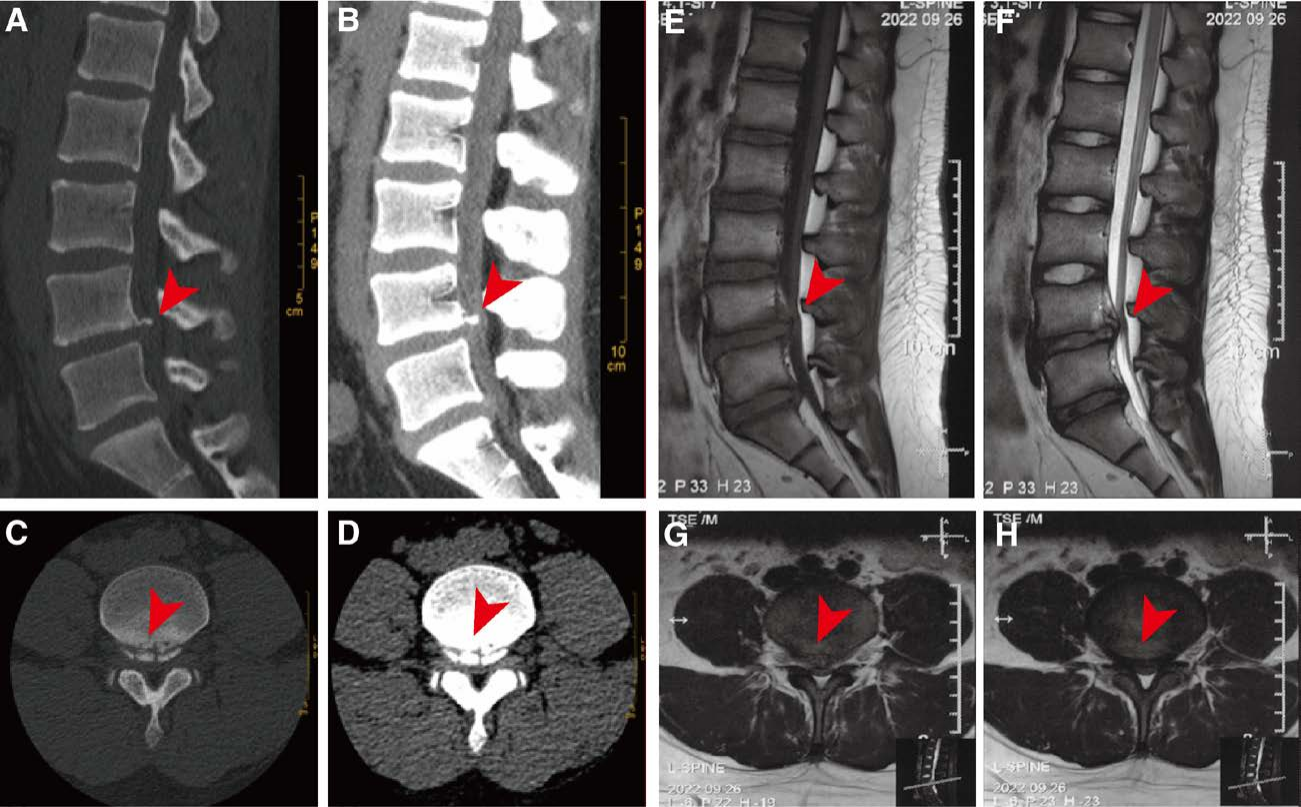
Pre-operative lumbar spine CT and MRI image features. (A–D/E and F) Pre-operative lumbar CT/MRI sagittal and axial planes showed L4-5 disc herniation (central type), fragments of PRAF with LDH at the lower L4 vertebral body were clearly visible, bilateral L5 nerve roots and the corresponding dural sac were compressed, and the spinal canal and bilateral lateral recess were both stenoses. CT = computer tomography, LDH = lumbar disc herniation. MRI = magnetic resonance imaging.

On Sep. 28, 2022, a 15-year-old schoolboy was brought in by his parents to the spine surgery clinic of our hospital, the University of Hong Kong-Shenzhen Hospital, a tertiary general hospital in China. The patient reported symptoms of low back and leg pain for half a year, and bilateral lower limb pain and numbness for 3 months. The child was an overweight with a body mass index of 30.4 kg/m^2^ (103 kg/1.84 m^2^). Past medical history is healthy. He enjoyed playing basketball in his spare time in the past 9 months. After playing basketball 3 months ago, the symptoms of back and leg pain became aggravated suddenly, accompanied by radiating pain on the posterolateral thighs and the posterior calf, bilateral lower limb numbness, prolonged standing, aggravated pain during walking and weight-bearing. No intermittent claudication was reported. Conservative treatment plans such as physical rehabilitation, weight reduction, and oral analgesics treatment failed to relieve his symptoms. Physical examination: Lumbar and back tenderness and percussion pain, light touch, acupuncture and deep sensation were normal in both lower limbs. Mild limitation of lumbar motion, bilateral knee reflex (++), bilateral ankle reflex (++), Lasègue’s sign (SLR) in both lower limbs (40º+), bilateral femoral nerve stretch test (FNS) (−), PR (−), grade 5/5 muscle strength and tension in both lower limbs. Radiographic data: X-ray, CT (computer tomography) and MRI (magnetic resonance imaging): L4-5 LDH, with compression of the corresponding dural sac and adjacent L5 nerve roots, spinal canal stenosis, fragments of PRAF at the lower L4 vertebral body were clearly visible (Figs. 1 and 2). Based on the above findings, a diagnosis of LDH with PRAF was made. To do that, we performed PELD surgery and successfully excised the herniated disc and epiphyseal fragments. Symptoms were immediately relieved postoperatively with a wound of only about 7.0 mm. Discharged on the next day. No perioperative complications occurred. Moreover, the imaging and clinical outcomes were also more satisfactory during the post-operative 15 months outpatient follow-up. The surgical procedure is as follows.

## 3. Surgical procedure

**Figure 3.**
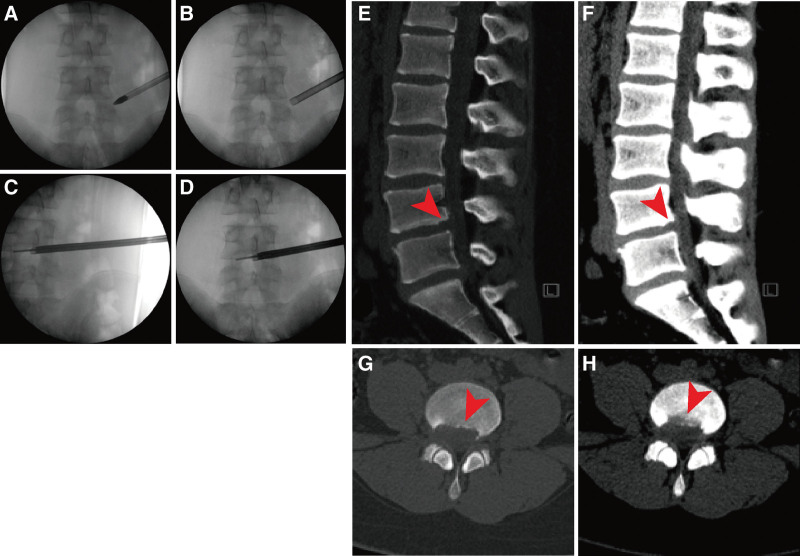
Pre-operative C-arm fluoroscopy and post-operative follow-up CT image at 6 wk. (A–D) Pre-operative C-arm fluoroscopy image. (E–H) Lumbar spine CT at 6 wk after PELD showed that the L4-5 disc herniation and PRAF fragments were completely removed, the corresponding dural sac, adjacent nerve root compression, and spinal canal narrowing were significantly reduced compared with the pre-operative. CT = computer tomography. PELD = percutaneous endoscopic lumbar discectomy, PRAF = posterior ring apophysis fracture.

After successful general anesthesia, the nerve monitoring electrodes were placed, laid prone on the carbon bed operating table, and operated with the transforaminal endoscopic spine system. Positioning the responsibility segment (L4-5) under the fluoroscopy of the C-arm (SIEMENS). Locating the responsibility level (L4-5) (Fig. 3A–D) under the fluoroscopy of the C-arm, and marking the right 12 cm next to the midline to locate the puncture needle point. Routine disinfection was performed, and an 18G puncture needle was placed at the puncture point, which glided along the lateral side of the superior articular process and entered the spinal canal through the safety triangular working zone of the intervertebral foramen. C-arm re-fluoroscopy confirmed that the puncture needle was located in the midline on the anterior radiographs and the posterior upper edge of the vertebral body on the lateral radiographs. We then inserted a guide wire to take out the puncture needle, cut the skin with a length of approx. 7.0 mm, inserted the expansion sleeve in turn to gradually expand the channel, installed the working channel and endoscope, and confirmed the position of the working channel through the C-arm again. Through endoscopic hemostasis and exploration, it can be seen that the disc herniation compresses the peridural nerve root. The protruding annulus fibrosus, nucleus pulposus and hyperplastic soft tissue were carefully removed with nucleus pulposus forceps and sent to pathology, and the lateral recess osteophyte was removed by grinding drill. Re-exploration under the endoscope showed that the nerve roots and dural sac were relaxed, and the surrounding decompression was complete. Then, porcine-derived fibrin adhesive (Bioseal Biotech Co., LTD., Guangzhou, China) was injected into the channel to stop bleeding and prevent bleeding. Drainage tube was indwelled in post-operative wound. Finally, re-fluoroscopy confirmed complete decompression of nerve roots and dural sac. Remove the working channel, suture and bandage the wound. Intraoperative bleeding was about 15 mL. No abnormality was found in nerve detection signals during the operation.

## 4. Discussion

LDH is a multifactorial condition that is the outcome of complex interplays among aging, genetics and the environment.^[20]^ LDH only accounts for about 1% of the total population, is common in adults, and has a low incidence in children. LDH is most common in the L4-5 and L5-S1 levels, and the mechanism remains unclear.^[21]^ Through a retrospective analysis of a Chinese cohort, Zhang et al found that family history, lumbar loading, hard physical labor, and time urgency were the major risk factors for LDH, and that physical exercise and sleeping on hard beds may be protective factors.^[22]^ Wang et al found that sedentary lifestyles may be one of the main risk factors for LDH among students.^[23]^ PRAF has been rarely described in previous reports on LDH, and its prevalence in the general population remains unknown. Because, PRAF is usually occult on plain radiographs, and it is likely that many spine surgeons or neurosurgeons are not familiar with this lesion and may be confused with the posterior longitudinal ligament, herniated disc calcification, and degenerative osteophytes. PRAF can occur in all vertebrae, usually at the same level as LDH, most commonly at the L4-5 and L5-S1 levels. This consistency may to some extent indicate the pathogenesis of PRAF and the close relationship in both of them.^[5,6,24,25]^ The outermost branch of the annulus fibrosa, Sharpey fibers, holds the disc in place with the adjacent vertebrae through the annulus process. The ring apophysis starts to gradually ossify at around the age of 6, fusing with the vertebral body by 17, before achieving complete fusion between 18 and 25. However, before the fusion is complete, there is a relatively weak link between the ring apophysis and vertebra.^[26]^ In addition, PRAF often occurs before the complete fusion of the ring apophysis, mainly at the superior or inferior edge of the vertebra, may be associated with acute effects from direct trauma or a history of related sports injuries, and more prevalent in overweight or obese children.^[25,27]^ In children, Keller and Lippitt et al have proposed 2 injury mechanisms: lumbar hyperextension and rapid flexion together with axial compression to the spine.^[28,29]^ Dietemann et al found through imaging that about 38% of young patients suffered from Scheuermann disease, resulting in epiphyseal ring degeneration.^[30]^ PRAF is more common in boys than girls, which may be related to boys’ preference for sports activities, such as physical exercise, including various ball games, dancing, gymnastics, skiing, diving, cycling, high jump, weightlifting, and wrestling, etc.^[24]^

It has been reported that X-ray, CT and MRI can be used to diagnose PRAF, but fracture or separation of ring apophysis is usually difficult to distinguish between plain X-ray and MRI imaging alone. In addition, axial CT is easily confused with ossification of the posterior longitudinal ligament or calcification of LDH and posterior degenerative ridge osteophytes, while sagittal CT can better display the location, size and shape of RAF or displacement. Therefore, CT has always been regarded as the first choice for diagnosis.^[4,16,27,30,31]^ However, X-ray can help exclude congenital dysplasia, Scheuermann disease, Schmorl node, abnormal vertebral body sequence, etc. MRI can more clearly show the impact of LDH on the surrounding dural sac, lateral recess or nerve root compression. Both of them, together with CT, guide the formulation of surgical or intervention treatment plan.^[27]^ Takata and Epstein et al divided PRAF fractures into 4 types: Type I: Simple separation of the entire posterior vertebral margin. Type II: Partial avulsion fracture of the vertebral body, including avulsion fracture of some of the substance of the vertebral body, including the margin. Type III: More localized lateral fracture of the posterior margin of the vertebral body. Type IV: Fracture that extends both beyond the margins of the disc and the full length of the vertebral body between the end plates.^[25,32]^ Takata et al found that the first 3 types of PRAF were usually not related to SLR (+), and only 1/4 are related to SLR (+). Therefore, when children have SLR (+) and imaging suggests PRAF, we should try to carefully look for the source of the symptoms.^[25]^ Because of the rarity and inexperience of LDH and PRAF in children, and the fact that they usually visit a pediatrician or neurologist first, the diagnosis time is usually delayed compared with adults.^[23,33]^

Through systematic literature search, we found that there is still no clear and unified diagnosis and treatment protocol for children with LDH or/and PRAF. Peul and Weber et al consistently found that early discectomy could relieve the clinical symptoms of adult patients with LDH faster than conservative treatment, but there was no significant difference between discectomy and conservative treatment after long-term comparison.^[34,35]^ In addition, approximately 20% to 50% of patients receiving conservative treatment require surgery at a later stage, but there is no evidence to show whether these conclusions could apply to children.^[34–37]^ Wu et al,^[24]^ through systematic literature review, argued that regardless of progressive nervous system dysfunction or intractable pain, patients should choose surgical treatment once their physical functions were affected. Otherwise, conservative treatment, including bed rest, brace fixation, physical therapy and non-steroidal anti-inflammatory drugs, etc, is recommended.^[38]^ Savini et al suggested that resection of herniated intervertebral disc cartilage and epiphyseal fragments could achieve satisfactory surgical results.^[39]^ Shirado et al found that if the epiphyseal fragment could not be moved during surgery, only the excision of the herniated disc could significantly relieve the compression of the affected nerve root, and the microscopic effect was almost the same as that of traditional discectomy. When the epiphyseal fragment can move, both the herniated disc and the epiphyseal fragment should be removed.^[40]^ LDH with PRAF are usually completely resected through traditional open surgery, but traditional surgery has disadvantages such as long incision, large trauma, heavy pain, large blood loss and long post-operative recovery time. Compared with traditional surgery, PELD (including transforaminal or interlaminar approach), an emerging minimally invasive spinal surgery, has the advantages of small incision, less trauma, less blood loss, faster post-operative recovery, and shorter hospital stay. However, both have almost the same clinical results and satisfactory results.^[41,42]^ In addition, Dabo and Chen et al found that the translaminar approach was more likely to damage the nerve root than the transforaminal approach, resulting in paresthesia in the lower limbs in the early post-operation, which may be related to the need to fully and thoroughly expose the field during the operation. Excessive stretching of the nerve root and dural sac also may be associated.^[43,44]^ For this reason, Chen et al introduced the PEAK method in PELD, which was to directly expose the calcified vertex and remove the calcified debris by increasing the amount of bone osteotomy and secondary forming of the foraminum, but it was still difficult to completely avoid the stretching of nerve roots and reduce the risk of nerve root injury.^[44]^ Therefore, we used PELD, based on the intra-operative experience of Dabo and Chen et al, successfully removed the herniated intervertebral disc and epiphyseal fragments step by step through the right transforaminal approach. The damage to the nerve root and dural sac was successfully avoided, and the patient was discharged home within 48 hours after the operation, achieving a very satisfactory result for the patient. Moreover, the imaging and clinical outcomes were also more satisfactory during the post-operative 15 months outpatient follow-up (Fig. 3E–H). Although PELD has been widely used in clinical practice, its role in pediatric LDH or/and PRAF remains limited. Lin and Wang et al reported that PELD achieved a very satisfactory post-operative effect with a very low complication rate.^[45,46]^ At the same time, it has the advantages of less trauma, short post-operative recovery time, short hospital stays, and small scar formation, which is a safe and effective treatment method for children with LDH.

## 5. Conclusion

Pediatric LDH with PRAF is extremely uncommon, and there is a lack of training among physicians for such cases, which may lead to delayed diagnosis and treatment. Here, it is recommended that when presented with cases of with low back pain or lower limb symptoms in the children or adolescents, physicians may conduct thorough examination first, including neurological examination, especially for those with overweight or a history of trauma, and carefully differentiate diagnosis based on the patient detailed history and CT imaging. Once a diagnosis for LDH with PRAF is established, PELD is a safe and effective minimally invasive treatment to be considered, and we hope that this technique can provide more assistance in the future.

## Acknowledgments

The authors would like to thank the patient for participating in this study.

## Author contributions

**Conceptualization:** Baode Zhang, Michael Kai-Tsun To, Kenneth Man-Chee Cheung, Jianbin Wu.

**Data curation:** Baode Zhang, Jianbin Wu.

**Formal analysis:** Baode Zhang, Peikai Chen, Kenneth Man-Chee Cheung.

**Funding acquisition:** Jianbin Wu.

**Investigation:** Baode Zhang.

**Methodology:** Baode Zhang, Jiaquan Zhong.

**Project administration:** Baode Zhang, Jianbin Wu.

**Resources:** Baode Zhang, Jianbin Wu.

**Software:** Baode Zhang.

**Supervision:** Baode Zhang, Michael Kai-Tsun To, Kenneth Man-Chee Cheung.

**Validation:** Baode Zhang.

**Visualization:** Baode Zhang.

**Writing – original draft:** Baode Zhang.

**Writing – review & editing:** Baode Zhang, Peikai Chen, Kenneth Man-Chee Cheung, Jianbin Wu.
